# Spaced education in medical residents: An electronic intervention to improve competency and retention of medical knowledge

**DOI:** 10.1371/journal.pone.0181418

**Published:** 2017-07-31

**Authors:** Jason Matos, Camille R. Petri, Kenneth J. Mukamal, Anita Vanka

**Affiliations:** 1 Clinical Fellow, Cardiovascular Division, Department of Medicine, Beth Israel Deaconess Medical Center, Boston, Massachusetts; 2 Resident, Department of Medicine, Beth Israel Deaconess Medical Center, Boston, Massachusetts; 3 Associate Professor of Medicine, Harvard Medical School, and Associate Program Director, Internal Medicine Residency Training Program, Department of Medicine, Beth Israel Deaconess Medical Center, Boston, Massachusetts; 4 Instructor in Medicine, Harvard Medical School, and Associate Program Director, Internal Medicine Residency Training Program, Department of Medicine, Beth Israel Deaconess Medical Center, Boston, Massachusetts; Pennsylvania State University College of Medicine, UNITED STATES

## Abstract

**Background:**

Spaced education is a novel method that improves medical education through online repetition of core principles often paired with multiple-choice questions. This model is a proven teaching tool for medical students, but its effect on resident learning is less established. We hypothesized that repetition of key clinical concepts in a “Clinical Pearls” format would improve knowledge retention in medical residents.

**Methods:**

This study investigated spaced education with particular emphasis on using a novel, email-based reinforcement program, and a randomized, self-matched design, in which residents were quizzed on medical knowledge that was either reinforced or not with electronically-administered spaced education. Both reinforced and non-reinforced knowledge was later tested with four quizzes.

**Results:**

Overall, respondents incorrectly answered 395 of 1008 questions (0.39; 95% CI, 0.36–0.42). Incorrect response rates varied by quiz (range 0.34–0.49; p = 0.02), but not significantly by post-graduate year (PGY1 0.44, PGY2 0.33, PGY3 0.38; p = 0.08). Although there was no evidence of benefit among residents (RR = 1.01; 95% CI, 0.83–1.22; p = 0.95), we observed a significantly lower risk of incorrect responses to reinforced material among interns (RR = 0.83, 95% CI, 0.70–0.99, p = 0.04).

**Conclusions:**

Overall, repetition of Clinical Pearls did not statistically improve test scores amongst junior and senior residents. However, among interns, repetition of the Clinical Pearls was associated with significantly higher test scores, perhaps reflecting their greater attendance at didactic sessions and engagement with Clinical Pearls. Although the study was limited by a low response rate, we employed test and control questions within the same quiz, limiting the potential for selection bias. Further work is needed to determine the optimal spacing and content load of Clinical Pearls to maximize retention amongst medical residents. This particular protocol of spaced education, however, was unique and readily reproducible suggesting its potential efficacy for intern education within a large residency program.

## Introduction

Internal medicine residency curricula are designed around six core competencies, designated by the Accreditation Council for Graduate Medical Education, that are required for graduation, including patient care, medical knowledge, professionalism, systems-based practice, practice-based learning, and communication skills [1]. Residents reach many of these competencies through direct patient care on inpatient and outpatient teaching services [2,3]. Medical knowledge in particular must be supplemented with small- and large-group didactic sessions. However, these sessions are often sandwiched between busy days of patient care, leaving little room for reflection and retention of subject matter.

Knowledge retention among medical housestaff remains a pervasive concern [4]. Repetition of key clinical concepts has been shown to improve retention of medical knowledge. Much of this research has involved spaced education (SE), a novel, evidence-based form of online education demonstrated to improve knowledge acquisition in randomized trials [4–6]. The SE protocol classically involves a spacing effect and testing effect. The spacing effect includes repeated exposure to medical knowledge over a given time period to reinforce and consolidate retention. The testing effect typically leverages the principle that testing improves a learner’s performance. Previous studies have shown this model is successful with medical students, pediatric residents and surgical trainees [4, 7–9].

We sought to determine whether a modified version of SE focused on the spacing effect could be applied to a novel, email-based reinforcement program on medical facts amongst medical house-officers, using a randomized, self-matched design in which interns and residents were tested on material that was or was not further reinforced at random with electronically-administered SE.

## Methods

The study was conducted at a large, single urban academic medical center with approximately 649 licensed beds. The internal medicine residency program is composed of approximately 160 housestaff. As education research, the project was deemed exempt from IRB approval by the Beth Israel Deaconess Medical Center Committee on Clinical Investigations.

To maximize retention, the residency program previously instituted a series of weekly "Clinical Pearls", or key learning points, from lectures and didactic sessions given throughout the week. These pearls are sent to all medical housestaff via email weekly and cover topics from major lecture series (Case Conferences, Morbidity and Mortality conference, Grand Rounds, Noon Conferences, Intern and Resident Report). All residents receive these “Pearls” each week, regardless of their individual rotation and attendance at conference. The Pearls document, which is drafted each week by a resident during his or her teaching elective, is typically one to two pages in length, in bullet-point format, and organized by day of week and didactic session.

Prior to this study, a survey of housestaff showed that 22% of residents read these emails weekly, while 72% read a Clinical Pearls email at least monthly. More than 60% of residents never referred back to old Clinical Pearls weekly emails. We hypothesized that synthesizing this learning material every month and testing this material would increase readership and improve retention.

From December 2013 to May 2014, one author (JM) compiled all weekly Pearls emails over a calendar month. Each individual learning point, or Pearl, underwent simple randomization to be reinforced or not reinforced via SE. Pearls that were not reinforced underwent no further repetition (i.e., the standard of care within the program). The reinforced Pearls were incorporated into a larger monthly document organized by subspecialty. This document was emailed each month to all housestaff in both Microsoft^®^ Word and Portable Document Format formats. The document contained reinforced Pearls from both the current and prior month (i.e., the February email contained reinforced Pearls from both January and February, etc.).

To test the spacing effect of the reinforced material, we sent out eight original multiple choice questions each month to the entire housestaff based directly on the Clinical Pearls. Half of these questions covered material that had been randomly reinforced by spaced learning in the weekly and monthly documents, and half covered material that had only appeared in the weekly email; these were not distinguished on the survey in any way (Table 1).

**Table 1:** Questions written each week.

	Clinical Pearl Reinforced	Clinical Pearl NOT Reinforced
Week 1	1 Question written	1 Question written
Week 2	1 Question written	1 Question written
Week 3	1 Question written	1 Question written
Week 4	1 Question written	1 Question written

Each week, after each individual pearl was randomized to SE or no SE, two questions were written by either a resident on their teaching elective or by the author (JM). One question pertained to clinical information to be reinforced by SE, and another question was based on a Pearl not reinforced by SE, for a total of eight questions. This strategy was repeated for five months.

The eight questions covered material from weeks one and three of the most recent month and weeks two and four of the month prior (Table 2), ensuring that all weeks’ lectures and Pearls were evaluated with questions.

**Table 2:** Composition of each quiz for housestaff.

Quiz	Content covered	Length
***Quiz 1*** **(After Month 2)**	**Week 2/4 Questions from MONTH 1 and Week 1/3 Questions from MONTH 2**	**8 Questions**
***Quiz 2*** **(After Month 3)**	**Week 2/4 Questions from MONTH 2 and Week 1/3 Questions from MONTH 3**	**8 Questions**
***Quiz 3*** **(After Month 4)**	**Week 2/4 Questions from MONTH 3 and Week 1/3 Questions from MONTH 4**	**8 Questions**
***Quiz 4*** **(After Month 5)**	**Week 2/4 Questions from MONTH 4 and Week 1/3 Questions from MONTH 5**	**8 Questions**

Each monthly eight question quiz included questions from the current month and the previous month. Four questions were taken from material randomized to SE and four questions were taken from material not randomized to SE.

Subjects responded anonymously to these questions. No questions were repeated across surveys, and answers were not provided until all responses to questions were collected. These multiple choice questions were written by a resident on their teaching elective or by the author (JM) based directly on the weekly Clinical Pearls emails. All questions were reviewed by two reviewers- (JM and AV) to ensure standardization of the level of difficulty, question format, and quality of the content tested. Questions were edited as necessary to meet the standards agreed upon between the two reviewers prior to study initiation. To maximize power, an incorrect response rate near but below 50% was sought.

In August 2014, during the subsequent academic year, a post-study survey was sent out to junior and senior residents who were housestaff during the study (S1 Fig). Using a Likert scale, the survey assessed how often housestaff read the weekly and monthly Pearls as well as the educational impact of the Pearls and quizzes. The residents were also asked to provide free text responses describing the barriers to reading Clinical Pearls and answering the quiz questions.

### Statistical methods

Because correct responses were more common than incorrect, we evaluated the likelihood of any given question being answered incorrectly as the primary outcome. We present the overall proportion of incorrect responses with exact binomial confidence intervals.

To evaluate the effect of reinforcement on the frequency of correct answers, we used generalized estimating equations to account for clustering of questions within-resident and within-quiz. We estimated the relative risk of an incorrect response to questions that had been reinforced with SE relative to those that had not, using a log link, binomial distribution, and exchangeable correlation matrix. We adjusted for post-graduate year (in three categories) except in analyses stratified by year. We tested the effect of post-graduate year and the specific quiz given similarly using type three tests with two or three degrees of freedom, respectively.

## Results

**Fig 1:**
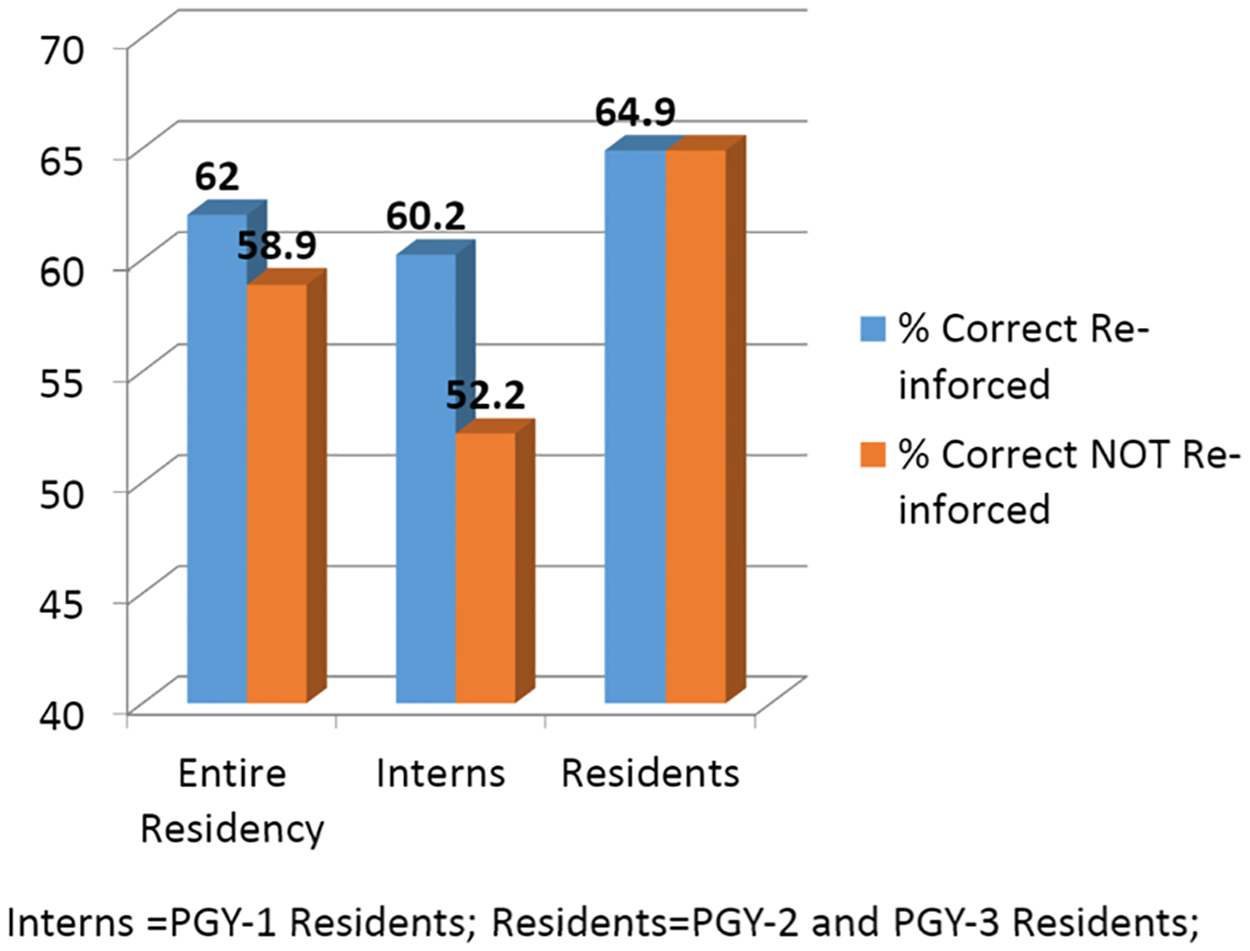
Quiz results on material reinforced and not reinforced. Quiz questions were more often correct on material that was reinforced, compared to on material not reinforced. Interns = PGY-1 Residents; Residents = PGY-2 and PGY-3 Residents.

A total of four quizzes, each comprised of eight questions, were distributed to housestaff. On average, 31 (SD 6.55) house officers responded to each quiz, representing a response rate of 20%. Of these, interns comprised 47% of respondents and junior and senior residents comprised the remainder. All respondents answered all the questions on each quiz, providing an identical number of questions to reinforced and unreinforced material. Overall, respondents incorrectly answered 395 of 1008 questions (39%; 95% confidence interval [CI], 36%-42%). Incorrect response rates varied by quiz (range 34%-49%; p = 0.02) but not significantly by post-graduate year (PGY) (PGY1 44%, PGY2 33%, PGY3 38%; p = 0.08). In crude analyses, 188 of 504 questions on reinforced material were answered incorrectly, compared with 207 incorrect responses to unreinforced material (Fig 1).

We next evaluated the relative risk of an incorrect response overall and among interns and residents. In analyses accounting for clustering and adjusted for post-graduate year, the relative risk of answering a reinforced question incorrectly compared with an unreinforced question was 0.90 (95% CI, 0.79–1.08; p = 0.14). Although there was no evidence of benefit among junior and senior residents (RR = 1.01; 95% CI, 0.83–1.22; p = 0.95), we observed a significantly lower risk of incorrect responses to reinforced material among interns (RR = 0.83, 95% CI, 0.70–0.99, p = 0.04) (Table 3).

**Table 3:** Relative risk of answering a question on reinforced (vs. unreinforced) material incorrectly.

Class	RR	95% CI	P-value
All housestaff	.90	.79–1.03	.14
**PGY-1**	**.83**	**.69-.99**	**.04**
PGY2 and PGY3	1.00	.82–1.22	.95

RR = Relative Risk; CI-Confidence Interval. Data was clustered by individual, month of quiz and adjusted for PGY class.

A total of 27 housestaff responded to the post-study survey (S1 Fig). Of these, 73% of responders felt the addition of the monthly Clinical Pearls helped them learn. However, 56% wished for immediate feedback to question responses and 48% felt that the reinforcement program with reminder messages involved too many emails.

## Discussion

In this randomized trial of a focused form of SE tied to an existing email system to reinforce key teaching points from weekly lectures, we found no statistical difference in retention of reinforced versus unreinforced material among our housestaff overall. However, retention was significantly better with reinforced material among interns.

There are several reasons that interns may have been most likely to respond to this intervention. Interns may have a steeper learning curve and therefore may be more motivated to review Clinical Pearls emphasized in didactic sessions, as more of this material is likely to be new. Indeed, their rate of incorrect responses was modestly higher than that of junior and senior residents, although residents of all years appeared to find the questions challenging (as intended). In addition, interns may be less likely to suffer from email fatigue, a common problem with this type of intervention in an increasingly digitalized housestaff environment. A post-survey from this project concluded that many interns and residents were turned off by the reinforcement of Clinical Pearls due to the frequency of emails.

Repetition of clinical material has been shown to improve retention over time in other studies as well. A 2012 study of CPR skills and knowledge similarly showed that recertification status blunted the deterioration of knowledge-based skills (i.e. scene safety and EMS activation) over time. However, components requiring specific skills, such as chest marking, declined over time despite recertification status [10].

In traditional models of SE, when students submit an answer, the student is immediately presented with the correct answer and an explanation of the topic [7]. In addition to augmenting retention, regimented multiple-choice quizzes may also improve medical residents’ standardized testing results. Recent work has shown that a continuous 12-month multiple-choice testing program improved PGY-3 in-service training exam scores [11]. This, coupled with didactic exam attendance, may help combat declining American Board of Internal Medicine passing rates [12]. Although we did not formally evaluate the testing effect in our trial, our results suggest that quizzes supplemented with immediate feedback may be received warmly, as the majority of respondents wanted quizzes to contain answers. Nonetheless, for long-term sustainability, we tested a design that only required compilation and reinforcement of existing email-based teaching points. Future studies could contrast this method to one in which bullet points are reinforced by testing, rather than reiteration, to determine if the added burden of quiz creation would merit its incremental effort.

Our study has both strengths and weaknesses. A particular strength of this study was its method of randomization. We did not randomize house officers; in our experience, substantial crossover can occur in novel interventions that only some residents receive. Instead, the subject matter was randomized, thereby allowing the entire housestaff to participate in the program. This eliminated unintended crossover between groups and also allowed each quiz to serve as its own control, increasing the efficiency of the design. We studied approximately 16 weeks of reinforced material, allowing for a broad range of questions and a large sample size of questions. The SE was all administered electronically, which required 1–2 hours of organizational labor each week, although most of the effort inherent in this study was devoted to studying its effect rather than to the intervention itself. We suspect that as smartphone applications that can be harnessed for medical education before more ubiquitous, the process of SE import and extraction may simplify [13–16]. Importantly, this intervention was well-liked amongst the housestaff. Based on our post-intervention survey, 74% of house officers agreed the Clinical Pearls were a good learning exercise and 67% of house officers said they learned from the monthly quizzes, despite a one week delay in receiving the answer key. We feel this particular protocol of spaced education, though unique, is easily reproducible and feasible even within a large and busy medical residency program. Barriers include the necessity for motivated and experienced residents to compile Clinical Pearls and organize quizzes. In our program, a dedicated house officer on a teaching elective greatly facilitated the protocol.

Limitations of this study include a low response rate. The multiple choice quizzes were optional and were given online, with the link provided via email. However, the fact that each quiz included both intervention and control questions substantially minimizes the bias that a low response rate might otherwise produce. In addition, we had no means of determining who read the weekly or monthly Pearls. Therefore, it was unclear whether those who read the Pearls were the same residents who took the quizzes; if not, the benefit of this intervention may have been markedly underestimated. This study also did not use a typical SE protocol. In our study, housestaff were not given immediate feedback to their quiz responses, and questions were not repeated in a regimented program as is typically performed with SE. Our effect size may have been stronger had we used a more quintessential model, albeit with less immediate applicability to programs like ours.

We have no objective measure of the quality of our assessment questions, although they were all edited by two co-authors, including an associate residency program director. Two observations suggest that these questions achieved their desired aims. First, we aimed for a correct-response rate similar to that measured, suggesting that our calibration of difficulty was accurate. Second, the fact that we observed an effect of SE using these questions, albeit only among interns, suggests that the questions were sufficiently sensitive to change; had they been substantially easier or more difficult, it would have been impossible to detect such an effect.

In summary, in this randomized trial, we found that reinforcement of learning material statistically improves medical retention among medical interns, but not medical residents. SE appears to be a promising and readily incorporated modality for improving retention when added to low-cost, highly-accessible electronic reinforcement systems.

## Supporting information

S1 Fig Survey to housestaff. Clinical Pearls Assessment Survey.
(TIFF)

S2 Fig IRB determination message. IRB determination email.
(MSG)

S3 Fig IRB determination form. IRB determination form.
(DOCX)
