# Supplementary figures and images for: Spaced education in medical residents: An electronic intervention to improve competency and retention of medical knowledge
Source: PLoS One. 2017 Jul 31;12(7):e0181418. doi: 10.1371/journal.pone.0181418 (PMC5536283; doi:10.1371/journal.pone.0181418)

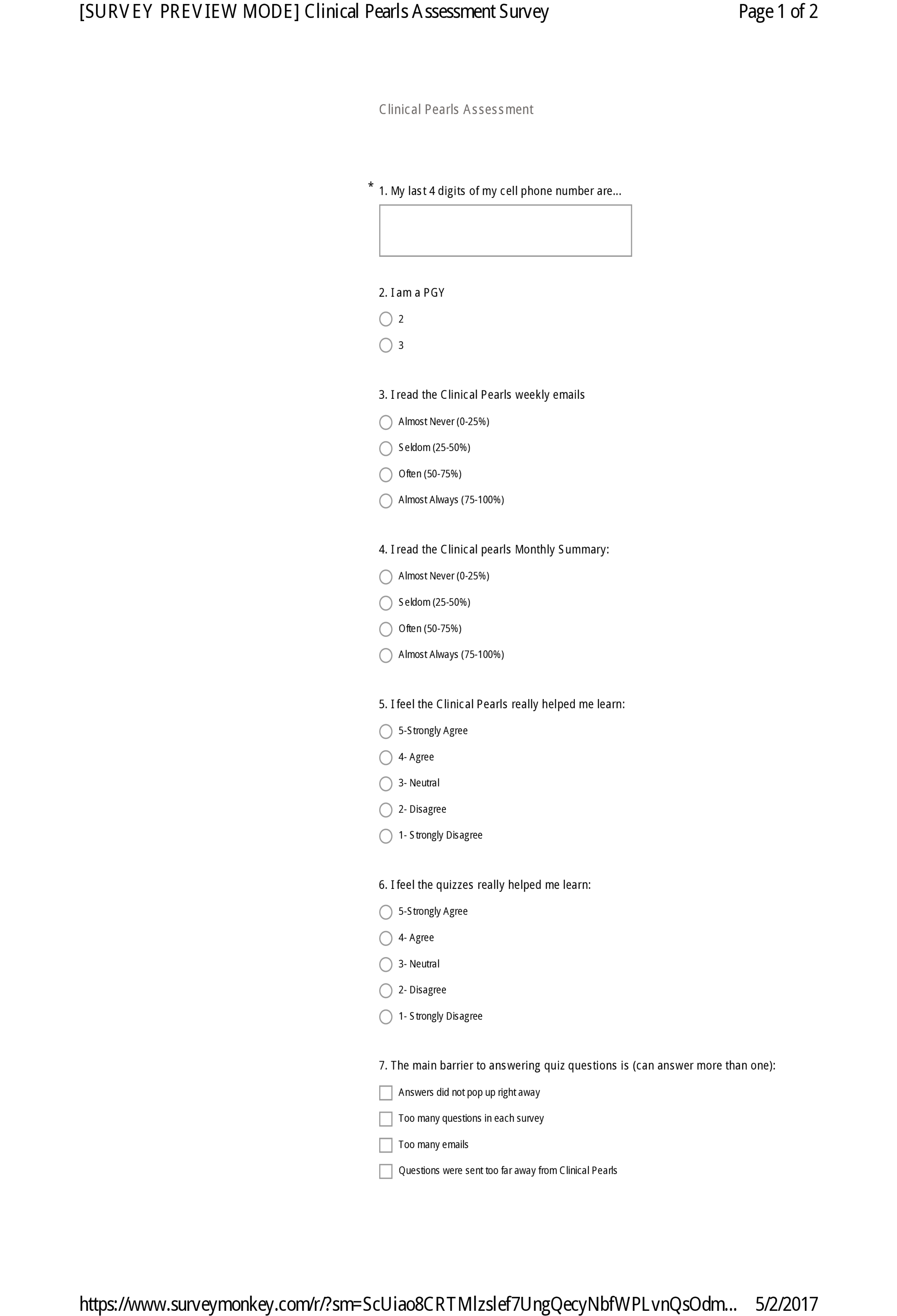

Supplement: S1 Fig — Clinical Pearls Assessment Survey. (TIFF) [file pone.0181418.s001.tiff]
