# Supplementary material for: Spaced education in medical residents: An electronic intervention to improve competency and retention of medical knowledge
Source: PLoS One. 2017 Jul 31;12(7):e0181418. doi: 10.1371/journal.pone.0181418 (PMC5536283; doi:10.1371/journal.pone.0181418)
Supplement: S3 Fig — IRB determination form. (DOCX) [file pone.0181418.s003.docx]

**DETERMINATION OF HUMAN SUBJECT RESEARCH**

All protocols involving both "research" or "clinical investigations" and "human subjects" must be reviewed and approved by the IRB before recruitment and data collection may start. The range of activities involving human **participants** at BIDMC comprises patient care, teaching and research; however, not all of these activities constitute human subject research. For example, training, education, quality improvement, and review of case reports are activities in which our faculty and staff are commonly engaged. However, for some activities it might be difficult to tell whether they qualify as human subject research. Furthermore, these activities may become research when an individual decides to take “accidental discoveries” and/or “innovative practices,” a step further and engage in a systematic investigation with the intent to contribute to generalizable knowledge.

Please refer to the CCI Policy Manual for further definition of human subject research.

<http://research.bidmc.harvard.edu/OST/CCI/Documents/CCIPolicyManual.doc>

If it unclear whether the activities in question meet the definition of “human subject research,” please complete the following questions and return the completed form as a Word document via email to Mary Williams, Director of IRB Operations at [mwillia2@bidmc.harvard.edu](mailto:mwillia2@bidmc.harvard.edu) for a determination.

**PROVIDE A TITLE OF PROJECT**

| Implementation of Spaced Learning to Improve Medical Knowledge Retention in Residency. |
| --- |

**WHO IS INVOLVED WITH THE PROJECT**

| Name of individual leading the project: Jason Matos MD | | | |
| --- | --- | --- | --- |
| Department: Internal Medicine | | Division: General Medicine & Primary Care | |
| Telephone: 617-632-8273 | Pager:93387 | | Fax: 617-632-0215 |
| Do you have other “collaborators” working on the project with you? If so, can you identify them at this time?  Anita Vanka MD (Mentor) | | | |

**EVALUATION**

| Provide a brief description (one paragraph) of the purpose or goal of the project, the intent for conducting the project,  and the procedures used to accomplish the purpose or goal:  The goal of this project is to determine whether the addition of spaced learning to an Internal Medicine Residency improves retention of medical knowledge presented during educational lectures. The intent is to improve the quality of medical education delivered at Beth Israel Deaconess Medical Center. Currently, weekly Clinical Pearls are disseminated via email to housestaff that highlight key lecture points from the week. A baseline needs assessment determined that the majority of residents do not read or refer back to these weekly pearls. Our hypothesis is that material that is presented and re-presented at weekly and monthly intervals respectively, will be better retained than material that is presented only weekly. To test our hypothesis, housestaff will receive monthly clinical pearls that will contain only half of the information covered in that month. These monthly clinical pearls will be sent to the Housestaff in a PDF format and also posted on the internal housestaff Wiki. Housestaff will then be asked to complete voluntary, anonymous multiple choice questions online which test the material included in the pearls from the prior 2 months as well as the material not included in the clinical pearls. Data will be analyzed as a composite of overall performance on the tests (% of each question answered correctly).    Does the project include testing the safety and efficacy of a drug or device in a human subject?  Yes  No  Do you intend that the information you learn from the project to be generalizable beyond BIDMC and BIDMC processes and practices? The intent is that the activity is undertaken to contribute to generalizable knowledge, not to provide immediate and continuous improvement and feedback in the local setting. Generalizable knowledge applies beyond a specific time and location.  Yes  No  Will you collect data from living individuals through some type of intervention?  Yes  No –  I will be collecting data via the multiple choice tests which are voluntary and anonymous.  Will you interact in any way with a living individual?  Yes  No  If yes, describe: We will be administering monthly multiple choice exams to housestaff, the they will fill out voluntarily. Their responses to this test will be anonymous and there will be no punishment for not completing the tests.  Will you have access to individually identifiable information?  Yes  No  If yes, describe: |
| --- |

**REVIEWER DETERMINATION – FOR CCI USE ONLY**

| The activities described do not meet the definition of Human Subject Research.  This project appears to be QI intended to improve the education of housestaff at BIDMC.  More information is required in order to determine if the activities meet the definition of Human Subject Research. Please provide the following information:  This project meets the definition of human subject research and should be submitted to the CCI as:  An Exempt Application  An Expedited Application  A Full Board Application  (Provide link to CCI Website for forms and instructions when returning a determination: <http://research.bidmc.harvard.edu/OST/CCI/CCIForms.asp> ) |
| --- |

Mary M. Williams, MS, CIP, Director, IRB Operations November 6, 2013

**Reviewer Name**  **Date**

**Signature**
